# *Inonotus obliquus* polysaccharide regulates gut microbiota of chronic pancreatitis in mice

**DOI:** 10.1186/s13568-017-0341-1

**Published:** 2017-02-14

**Authors:** Yang Hu, Chunying Teng, Sumei Yu, Xin Wang, Jinsong Liang, Xin Bai, Liying Dong, Tao Song, Min Yu, Juanjuan Qu

**Affiliations:** 1 College of Resources and Environmental Science, Northeast Agricultural University, Harbin, 150030 People’s Republic of China; 2 School of Life Sciences, Tsinghua University, Beijing, 100084 People’s Republic of China; 3 The Shenzhen Key Laboratory of Gene and Antibody Therapy, State Key Laboratory of Health Science and Technology (prep), Center for Biotechnology & Biomedicine and Division of Life & Health Sciences, Graduate School at Shenzhen, Tsinghua University, Shenzhen, 518055 People’s Republic of China; 4 Drug Safety Evaluation Center, Heilongjiang University of Chinese Medicine, Harbin, 150040 People’s Republic of China

**Keywords:** *Inonotus obliquus* polysaccharide, Chronic pancreatitis, Mice, Gut microbiota, High throughput sequencing

## Abstract

Polysaccharide is efficient in attenuation of metabolic ailments and modulation of gut microbiota as prebiotics. The therapeutic effect of *Inonotus obliquus* polysaccharide (IOP) on chronic pancreatitis (CP) in mice has been validated in our previous study. However, it is not clear whether IOP is conducive to maintaining the homeostasis between gut microbiota and host. The aim of this study is to testify the potential effects of IOP on gut microbiota composition and diversity in mice with CP. The changes in glutathione peroxidase (GSH-P_X_), total antioxidant capacity (TAOC), tumor necrosis factor alpha (TNF-α), transforming growth factor beta (TGF-β), lipase and trypsin levels were measured by commercial assay kits, meanwhile the gut microbiota composition and diversity were analyzed by high throughput sequencing. The IOP treatment increased GSH-P_X_ and TAOC levels, and decreased TNF-α, TGF-β, lipase and trypsin levels in CP mice. It was also observed that gut microbiota in IOP treated groups were less diverse than others in terms of lower Shannon diversity index and Chao 1 estimator. IOP increased the proportion of *Bacteroidetes* and decreased that of *Firmicutes* at phylum level. *Bacteroidetes* was found positively correlated with GSH-P_X_ and TAOC, and *Firmicutes* correlated with TNF-α, TGF-β, and lipase. In conclusion, administration of IOP could regulate gut microbiota composition and diversity to a healthy profile in mice with CP, and some bacterial phylum significantly correlated with characteristic parameters.

## Introduction

Chronic pancreatitis (CP) is a progressive and permanent destruction of the pancreas leading to insufficient exocrine and endocrine, and often chronic disabling pain. The complications of CP commonly include diabetes mellitus, cholangitis, ascites and even carcinoma of pancreas (Inui et al. 2013; Phillips 2012). In developed countries, CP incidence ranges from 3.5 to 10 per 100,000 populations, while the unhealthy lifestyle has caused a gradual rise in CP (Witt et al. 2007). Nowadays in China the incidence of CP is about to surpass 1% which is 20 times higher than that in 1950s. The consumption of alcohol as well as genetic and environmental factor might cause CP (Braganza et al. 2011). Currently, oxidative stress has been implicated as a potential mechanism in etiology and pathology of CP (Zhou et al. 2015). 3, 5-Diethoxycarbonyl-1,4-dihydrocollidine (DDC) is a kind of SOD inhibitor that may cause oxidative damage and following fibrosis in pancreas (Matsumura et al. 2001). DDC has been managed to induce CP in mice (Matsumura et al. 2001; Fickert et al. 2014).

Under long-term oxidative stress, the invasion of pathogenic bacteria can destroy the microenvironment, disturbed the structure of intestinal flora, and ultimately lead to dysbiosis (Burcelin et al. 2011; Ley et al. 2008a, b). The correlation between the variation of gut microbiota and the development of bowel inflammatory, diverticulitis, diabetes, obesity etc. has been explicit, but it remains elusive as for CP (Rautava and Isolauri 2002; Berry et al. 2012; Daniels et al. 2014; He et al. 2015; Lim et al. 2015). Recently, Tan et al. reported the relationship between intestinal microbiota composition and inflammation involved in the progression of acute pancreatitis (Tan et al. 2015). Although the mechanism involved in these changes has not been fully elucidated, a major shift in gut microbiota composition was found in patients with severe and mild acute pancreatitis, especially an increasing relative abundance of *Enterococcus* and a decrease of *Bifidobacterium*.

Polysaccharides contribute to the proliferation of “good bacteria” and regulate microbial composition in host gut. Several polysaccharides like lentinan, glucans, mannans and xylans have the prebiotic effects on the intestines such as increasing the resistance of intestinal mucosa to inflammation and inhibiting the development of intestinal ulcers in rats (Singdevsachan et al. 2016). Shi et al. also found that a water-soluble-d-fructan extracted from the roots of *Ophiopogon japonicas* exhibited potent anti-obesity and hypoglycemic effects via regulating the gut microbiota of the host as prebiotics (Shi et al. 2015). *Inonotus obliquus* (also called Chaga), is a white-rot basidiomycete parasitizing on *Betula* (birch) trunks in low latitudes (about 45–50°N) of Europe, Asia and North America. Experiments showed that *I. obliquus* could be used as drugs to prevent and cure cancer, diabetes, cerebrovascular diseases, etc. (Zhou et al. 2015; Ma et al. 2013). *Inonotus obliquus* polysaccharide (IOP) possesses comprehensive biological properties, especially antioxidant and anti-inflammatory activities. As a superior antioxidant, IOP has been used to attenuate CP in our previous research (Hu et al. 2016). However, the overall gut microbiota structure and diversity in CP mice administrated with IOP was ambiguous.

To verify the relationship between CP and gut microbiota, a clinical experiment in mice was conducted to evaluate the variations of glutathione peroxidase (GSH-P_X_), total antioxidant capacity (TAOC), tumor necrosis factor alpha (TNF-α), transforming growth factor beta (TGF-β), lipase, trypsin and the overall changes of gut microbiota in feces. The discovery of their correlation may be contributed to CP pathogenesis and therapy in terms of the intestinal ecosystem.

## Materials and methods

### Chemicals and reagents

*Inonotus obliquus* (CFCC83280) was provided by Harbin Baykaltai Bioengineering Co. LTD of China. Anion-exchange DEAE cellulose column and Sephadex G-200 gel were obtained from Pharmacia (USA). Chloroform, butanol and ethanol were purchased from Kermel Chemical Reagent Co. (Tianjin, China). DDC was purchased from Sigma Chemical Co. (St Louis, USA). All solutions were prepared by analytical reagents and double distilled water. GSH-P_X_, TAOC, TNF-α, TGF-β, lipase and trypsin were detected by commercial assay kits purchased from the Nanjing Jiancheng Bioengineering Institute (Nanjing, China). DNA mini stool kit was purchased from Qiagen (Valencia, CA, USA). MetaVx™ library preparation kit was purchased from GENEWIZ Institute (South Plainfield, NJ, USA).

### Preparation of IOP

IOP was prepared according to our previous procedure (Hu et al. 2016). Briefly, the dried sclerotia of *I. obliquus* were ground to powder and extracted with distilled water at 60 °C for 2.5 h. The supernatant was concentrated and treated with Sevag reagent (Chloroform: butanol = 5:1) to remove protein. Then the supernatant was mixed with four volumes of 95% ethanol and kept at 4 °C for 12 h. Crude IOP was centrifuged, lyophilized and further purified in an anion-exchange DEAE cellulose column (50 cm × 2.6 cm) which was eluted with 0.05 M, 0.1 M and 0.2 M NaCl solution and a Sephadex G-200 gel column (1.6 cm × 40 cm). The obtained IOP is a homogeneous polysaccharide with molecular weight of 32.5 kDa, polysaccharide content of 98.6%, and monosaccharide composition of Man, Rha, Glu, Gal, Xyl and Ara in a molar ratio of 9.8:13.6:29.1:20.5:21.6:5.4 (Hu et al. 2016).

### Toxicity test

Toxicity test was performed according to our previous study (Hu et al. 2017). Briefly, specific pathogen-free male ICR mice (18–22 g) were purchased from Drug Safety Evaluation Center of Heilongjiang University of Chinese Medicine. IOP was administrated to mice at a dose of 1 g/kg body weight by oral gavage three times a day. Control group received saline solution. Sterilized water and standard chow were provided for all mice. The mortality and side effects of mice were observed for 72 h.

### Experimental design

Mice were randomly divided into six groups with ten mice in each group: three IOP treated groups (IOP-L, IOP-M and IOP-H), Qingyilidan granule treated group (PC), model control group (MC), and normal control group (NC). Except for mice in NC group, all mice were received intraperitoneal injections with DDC (10%, 0.5 g/kg body weight) twice a week for continuous four weeks (Fickert et al. 2014). Mice in IOP treated groups were orally administrated with IOP at a dosage of 0.1 (IOP-L), 0.2 (IOP-M) and 0.4 g/kg/day (IOP-H) body weight for continuous four weeks from the second week of the DDC injection, respectively. PC group was daily fed with Qingyilidan granule at a dose of 3.7 g/kg/day. Mice in MC and NC groups were given normal saline to osmotic pressure of 0.9%. All mice were kept in a 12 h dark/light cycle room with humidity-control at a constant temperature of 25 °C. They all had free access to sterilized water and standard chow. After 5-week experiment, all mice were scarified via cervical dislocation.

### Measurement of GSH-Px, TAOC, TNF-α, TGF-β, lipase and trypsin

The pancreas tissue was homogenized with normal saline by sonication. The activities of GSH-Px and TAOC were determined using commercial kits, and they were expressed as U/mg wet weight of pancreatic tissue.

Blood samples were collected from the eye vein by removing eyeball. Then serum was separated by centrifugation at 3000 rpm for 10 min at 4 °C and stored at −80 °C until use. The contents of serum TNF-α and TGF-β were determined by commercial assay kits according to manufacturer’s instructions. The activities of TNF-α and TGF-β were expressed as pg/mL.

Serum lipase and pancreatic trypsin were quantified using detection kits according to manufacturer’s instructions. Lipase and trypsin contents were expressed as U/L and pmol/mg respectively. Each sample was analyzed in triplicate.

### Preparation of genomic DNA from fecal samples

The fecal samples were collected from each group at the end of the experiment. Total DNA was extracted according to the method of Yu and Morrison (Yu and Morrison 2004). 0.5 g (wet weight) of fecal sample was suspended in 2 mL of breaking buffer (0.5 M NaCl, 50 mM Tris–HCl, 50 mM EDTA, 4% sodium dodecyl sulfate) at 70 °C for 15 min after shaking for 30 s. The mixture was centrifuged at 6000 rpm for 5 min at 4 °C and the supernatant was removed and retained. This procedure was repeated for 3 times and the obtained supernatants were pooled. Nucleic acids were extracted sequentially with ammonium acetate and isopropanol. They were then treated with DNase-free RNase, proteinase K, and further purified with DNA mini stool kit.

### MetaVx™ library preparation and Illumina MiSeq sequencing

Next generation sequencing library preparations and Illumina MiSeq sequencing were conducted at GENEWIZ, Inc. (Beijing, China). DNA samples were quantified using a Qubit2.0 Fluorometer (Invitrogen, Carlsbad, CA) and DNA quality was checked on a 0.8% agarose gel. 5–50 ng DNA was used to generate amplicons using a MetaVx™ library preparation kit. A panel of proprietary primers was designed to anneal to the relatively conserved regions bordering V3, V4, and V5 hypervariable regions. The V3 and V4 regions were amplified using forward primer in sequence of CCTACGGRRBGCASCAGKVRVGAAT and reverse primer in sequence of GGACTACNVGGGTWTCTAATCC. The V4 and V5 regions were amplified using forward primer in sequence of GTGYCAGCMGCCGCGGTAA and reverse primer in sequence of CTTGTGCGGKCCCCCGYCAATTC. All PCR products were purified with the QIAgen DNA Mini Stool Kit. DNA libraries were validated using an Agilent 2100 Bioanalyzer (Agilent Technologies, Palo Alto, CA, USA), and quantified by Qubit and real time PCR (Applied Biosystems, Carlsbad, CA, USA). DNA libraries were multiplexed and loaded on an Illumina MiSeq instrument according to manufacturer’s instructions (Illumina, San Diego, CA, USA) by GENEWIZ.

### Bioinformatic and statistical analysis

The sequences were clustered into operational taxonomic units (OTUs) using a 97% identity cut-off. OTUs were used for diversity and richness analysis. Partial least square discriminate analysis (PLS-DA) was used to identify OTUs by Simca-P+ software (version 12.0, Umetrics AB, Umea, Sweden). Variable importance in projection (VIP) was used to select the key OTUs based on their contribution to the biochemical characteristics of our study. The OTUs with the highest contribution (VIP score >1.0) were translated to the key OTUs and used to clarify the relationships between groups and key OTUs. Pearson correlation analysis in SPSS 18.0 (SPSS Inc., Chicago, IL, USA) was used to evaluate the correlations between biochemical characteristics and gut microbiota at phylum level. Clustering analysis and heatmap were performed using Mothur and R software (http://www.mothur.org/wiki/MainPage). Canonical correspondence analysis (CCA) was determined by Canoco 4.5 (Biometrics, Wageningen, The Netherlands). Sequences used in this study were deposited to the NCBI Sequence Read Archive (accession number SRP067729).

Biochemical characteristics data were analyzed with SPSS 18.0. General characteristics were expressed as median and mean or percentages. Statistical analyses were carried out using one-way analysis of variance. Statistical significance was calculated by Student’s t test and a probability value *P* < 0.05 or *P* < 0.01 was considered to be significant in statistic.

## Results

### Toxicity test

During the experimental period, no death was observed. The mice treated with IOP did not show any side effects.

### Biochemical characteristics analysis of GSH-Px, TAOC, TNF-α, TGF-β, lipase and trypsin

**Fig. 1:**
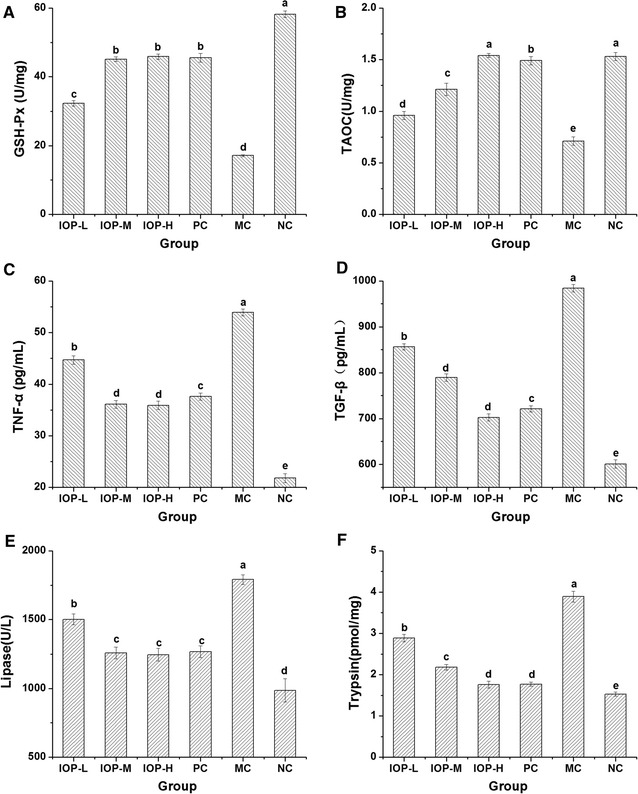
Effect of IOP on biochemical parameters in CP mice induced by DDC. **A** Effect on pancreas GSH-Px level. **B** Effect on pancreas TAOC level. **C** Effect on serum TNF-α level. **D** Effect on serum TGF-β level. **E** Effect on serum lipase level. **F** Effect on pancreas trypsin level. Values are shown as the mean ± SD (n = 10). Means within *each error bar* having *different letters* are significantly different (p < 0.05)

As an important enzyme in superoxide degradation, GSH-Px has been taken as an index to the antioxidative status in organism (Rotruck et al. 1973), and a decrease in GSH-Px level is generally observed in carcinoma, sclerosis and CP patients (Sehitogullari et al. 2014; Adamczyk-Sowa et al. 2012; Girish et al. 2011). The level of TAOC also can reflect the capacity of nonenzymatic antioxidant defense system (Li et al. 2007). As shown in Fig. 1A, B, the pancreatic GSH-Px and TAOC in MC group were lowest indicating that DDC inducement had caused pancreatitis in mice. The higher levels of GSH-Px and TAOC in all IOP groups than those in MC group (*P* < 0.01) illustrated that administration of IOP could prevent pancreas from oxidative damage. Moreover, IOP treatment on GSH-Px and TAOC presented a dose-dependently effect, the GSH-Px level of IOP-L, IOP-M and IOP-H group was 32.3 ± 0.8, 45.1 ± 0.7 and 45.9 ± 0.8 U/mg respectively, the TAOC level was 0.96 ± 0.04, 1.21 ± 0.06, and 1.54 ± 0.02 U/mg respectively. And the level of TAOC in IOP-H group was insignificantly different from that in NC group (*P* > 0.05).

Levels of serum TNF-α and TGF-β in mice at the end of experiment were given in Fig. 1C, D. The value of TNF-α in MC group was 53.9 ± 0.7 pg/mL which was about 2.5 times higher than that in NC group (21.8 ± 0.9 pg/mL) (*P* < 0.01), indicating that the prolonged inflammatory response in CP mice might result in an increase in the systemic concentration of TNF-α (Petersen and Pedersen 2005). In IOP-L, IOP-M, IOP-H and Qingyilidan granule group, TNF-α level in mice was about 22.9, 14.3, 14.1 and 15.8 pg/mL higher than that in NC group respectively, but greatly lower than that in MC group (*P* < 0.01). Likewise, the level of TGF-β in MC group increased 383.04 pg/mL compared with NC group (*P* < 0.01), indicating that DDC inducement had caused the injury of pancreas in mice (Schneider et al. 2004). However, the levels of TGF-β were significantly decreased in the IOP and PC groups when compared with MC group.

Data on serum lipase and pancreatic trypsin levels in mice at the end of the experiment were presented in Fig. 1E, F. The highest level of lipase was found in MC group which was 1.2, 1.4, 1.4, 1.4 and 1.8 times higher than that in IOP-L, IOP-M, IOP-H, PC and NC group respectively. Benini et al. found that an increase in lipase level was always detected in patients with CP (Benini et al. 1987). The curative effect of IOP was not invariably dose-dependent, just as the high and moderate dose of IOP had a same effect on lipase (*P* > 0.05). Similarly, activity of trypsin in MC group was higher than that in NC group. However, IOP and Qingyilidan granule decreased the level of trypsin and the insignificant difference between PC and IOP groups (*P* > 0.05) suggested that activity of IOP amounted to the commercial herb medicine that is generally used for CP therapy.

### OTU, cluster analysis, diversity and richness of six groups

For six groups, variable regions (V3–V5) of the bacterial 16S rRNA gene were amplified by PCR. From all the fecal samples, a dataset consisting of 409,812 high-quality 16S rRNA gene sequences was obtained (Table 1). A total of 47,950 OTUs were identified based on the conventional criterion of 97% similarity (equal to species level), more specifically, 9652 OTUs for IOP-L group, 9439 OTUs for IOP-M group, 5918 OTUs for IOP-H group, and 6903 OTUs for PC group, 10815 OTUs for MC group, 5223 OTUs for NC group with an average sequence length of 298 bp.

**Table 1:** Illumina MiSeq sequencing data

	Seq_num	OTU	Shannon diversity index	Chao 1 estimator
IOP-L	80,150	9652	8.71	45,078.72
IOP-M	75,410	9439	8.69	39,521.43
IOP-H	53,515	5918	8.44	23,794.53
PC	66,853	6903	8.50	33,496.84
MC	90,192	10,815	9.00	57,089.69
NC	43,692	5223	8.24	20,227.70

**Fig. 2:**
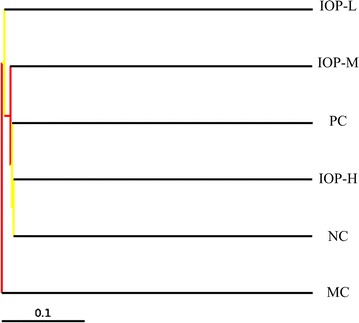
Cluster analyses of gut microbiota in different treatments. *Dendrogram* indicates six groups are divided into two branches. IOP-H, IOP-M, IOP-L and PC groups cluster in one branch with NC, but MC group is an independent branch which has low similarity with others

Cluster analysis using unweighted pair group method with arithmetic mean (UPGMA) was shown in Fig. 2. Based on cluster analysis for gut microbiota structure, an obvious difference was shown among the six groups which were consistent with the divergence of biochemical characteristics. Genetic distance of cluster analysis showed that the six groups could be divided into two branches. IOP-H, IOP-M, IOP-L, PC and NC groups clustered in one branch with IOP-H group on the first grade. MC group was categorized as an independent branch, indicating a low similarity with others.

Shannon diversity index and Chao 1 estimator were used to describe the diversity and richness of microbiota. In this study, gut microbiota diversity and richness were significantly increased by DDC inducement as presented in MC group (Table 1). For three IOP groups, Shannon diversity indexes were lower than that of MC, but higher than that of NC. Chao 1 estimator of NC group was 20227.70, but that of MC group reached 57,089.69. The results indicated the ecological balance of gut microbiota was disrupted in CP mice. However, IOP and Qingyilidan granule treatments were conducive to maintaining the microbiota structure and richness to normal level as that in NC group. And with the increasing dose of IOP, the community richness was correspondingly declined.

### Correlation analysis

**Fig. 3:**
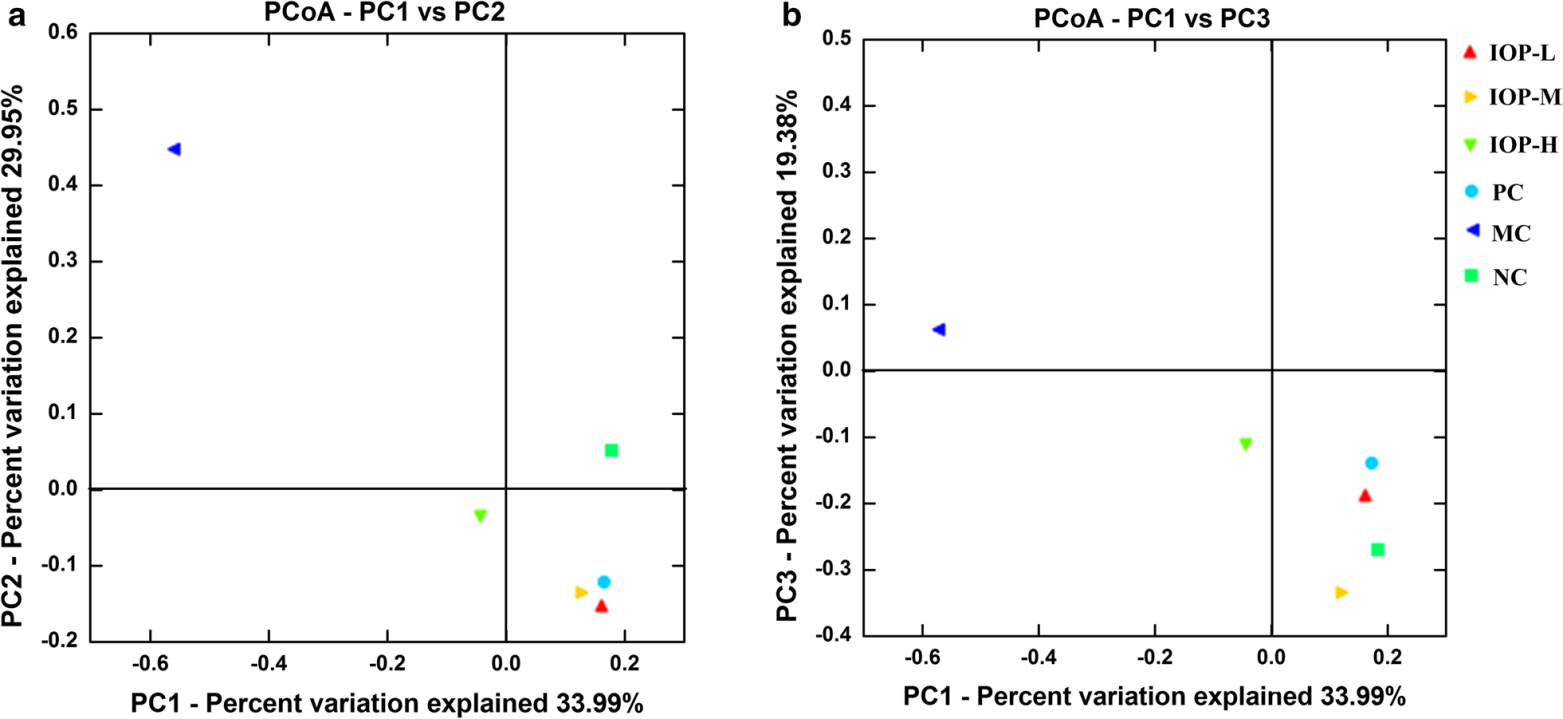
Unweighted principal coordinate analysis plots: Unweighted UniFrac PCoA plotted against PC1 versus PC2 axes (**a**) and PC1 versus PC3 axes (**b**). The *plots* show the clustering pattern among IOP-L, IOP-M, IOP-H, PC, MC and NC groups. MC group is far from the other five groups, and the *plots* indicate the change of clustering after DDC injection and IOP intake

Principal coordinates analysis (PCoA) is usually used for visualizing and analyzing the association of sample metadata, it can also reveal the divergency of bacterial community structure. The PCoA plots of six groups were shown in Fig. 3. Figure 3a displayed that all IOP, PC and NC groups assembled in the right area of the bottom, MC group scattered in another area. It indicated that gut microbiota in three IOP treated groups and PC group were relevant to that in NC group, but MC group far from other groups with PC1 accounting for 33.99% of the total variation (Fig. 3a). The gut microbiota in six groups also differed from each other along PC2 and PC3 (Fig. 3b). Three IOP groups were far from MC group along PC2 and PC3, and they were distinctively apart from NC group. Overall, IOP and PC groups were far from MC group, but near to the NC group, and the PCoA plot visualization explained over 83% of the microbiota variation. The results showed that the variation of gut microbiota structure was closely related to the degree of CP, and IOP could remarkably change the structure of gut microbiota.

**Fig. 4:**
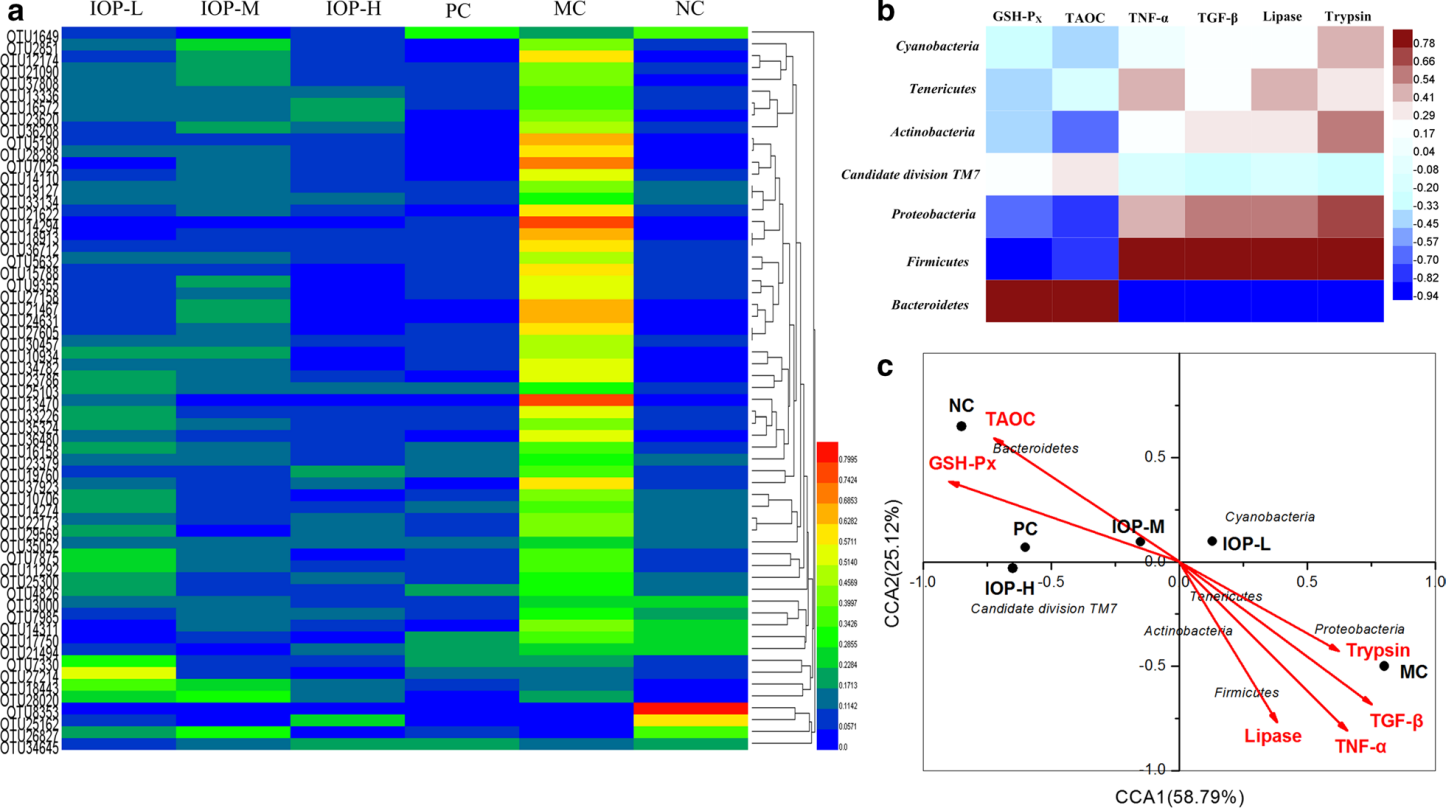
Correlation analysis. **a** Heatmap of key OTUs in the six groups. **b** Heatmap of biochemical parameters and gut microbiota at phylum level. **c** Canonical correspondence analysis (CCA) of six groups

61 Key OTUs were selected by PLS-DA with highest contribution to the biochemical characteristics (VIP score >1.0). The heatmap shown in Fig. 4a indicated the relative percentage of key OTUs in the six groups. The relative value was presented by the color intensity and the legend was illustrated in the figure. The structure of gut microbiota in IOP, PC and NC groups shared higher similarity compared with that in MC group.

Based on Pearson correlation analysis, the correlations of six biochemical parameters and gut microbiota at phylum level were shown in Fig. 4b. This plot revealed that *Bacteroidetes* (increased by IOP) had significant positive correlation with GSH-Px (P < 0.05) and TAOC (P < 0.05), while negative correlation with TNF-α (P < 0.01), TGF-β (P < 0.01), lipase (P < 0.05) and trypsin (P < 0.05). *Firmicutes* (decreased by IOP) was negatively associated with GSH-Px (P < 0.05), but positively associated with TNF-α (P < 0.05), TGF-β (P < 0.05), and lipase (P < 0.05).

Canonical correspondence analysis (CCA) of six groups was shown in Fig. 4c. 58.79% of the variations were explained by CCA1 while 25.12% were explained by CCA2. The biplot showed that gut microbiota was significantly shaped by GSH-Px, TAOC, TNF-α, TGF-β, lipase and trypsin. The results indicated that biochemical parameters and their interactions might be essential to the maintenance of gut microbiota structure. PC and IOP-H group was close to each other and they were not far from NC group. This result was in accordance with that of PCoA analysis.

### Variation of gut microbiota composition at phylum and genus level

**Fig. 5:**
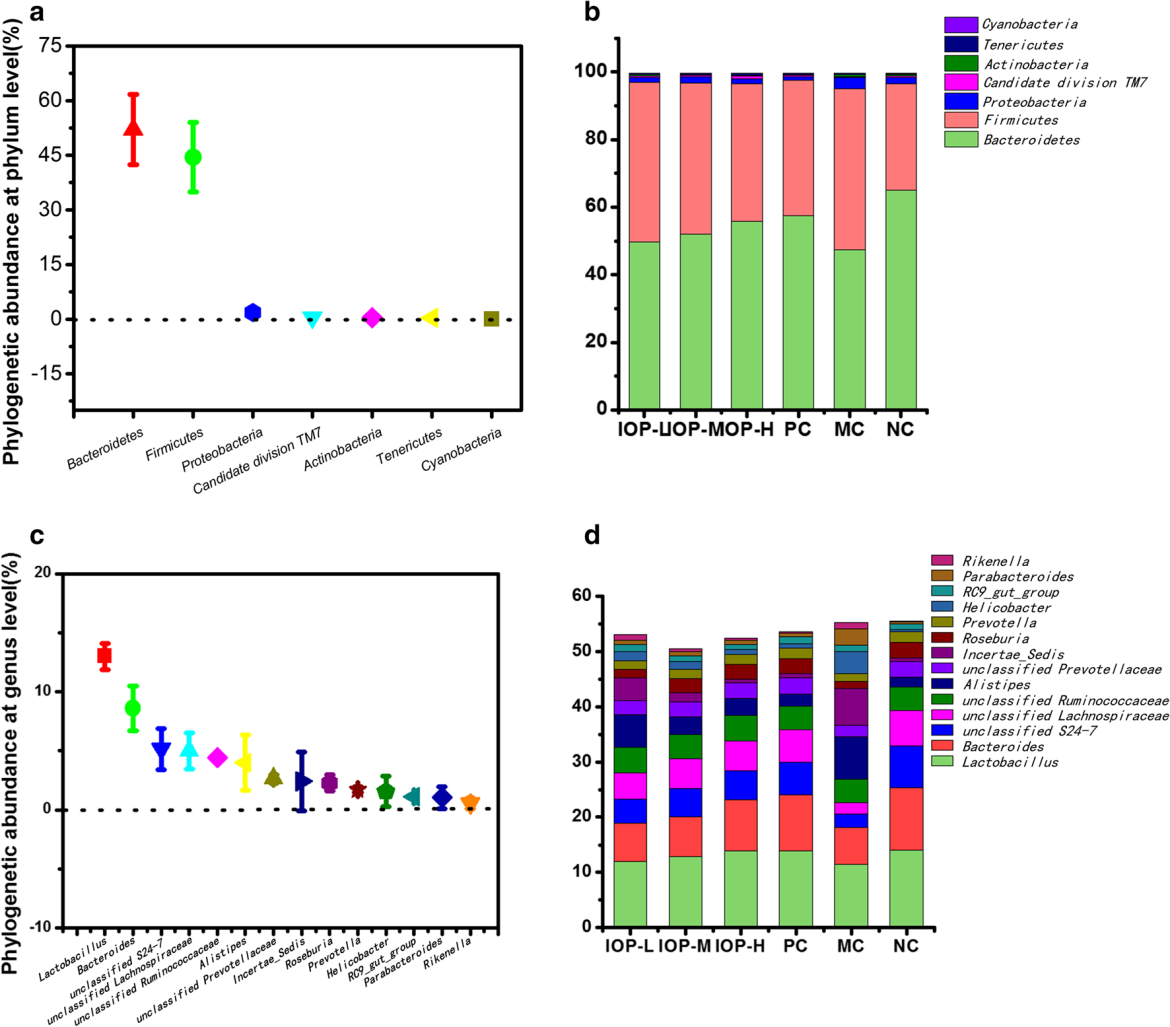
Relative abundance of gut microbiota at phylum and genus level. **a** Phylogenetic abundance at phylum level. **b** Dominant phyla in each group. **c** Phylogenetic abundance at genus level. **d** Dominant genera in each group

In order to explore the overall variation in gut microbiota composition among six groups, the community difference at phylum and genus level was compared. Based on the core OTUs of taxonomic database analyzed by program QIIME, a total of 7 major phyla were identified in the six groups (Fig. 5a). The predominant phylum was *Bacteroidetes*, which contributed 65.05% to the total sequence reads in NC group and 47.47% in MC group. However, the proportion of *Bacteroidetes* was increased about 4.55, 9.56, 17.48 and 20.81% in IOP-L, IOP-M, IOP-H and PC groups respectively, compared with that in MC group. *Firmicutes* was the subdominant phylum, which contributed 47.52% to the total sequence reads in MC group. Proportions of *Firmicutes* in IOP and Qingyilidan groups were all significantly lower than those in MC group, but higher than that in NC group. And the relative abundance of *Firmicutes* was decreased 14.46 and 15.57% in IOP-H and PC group compared with that in MC group. Other phyla including *Actinobacteria*, *Candidate division TM7*, *Cyanobacteria*, *Proteobacteria* and *Tenericutes*, together contributed a lower percentage to the total bacterial sequences. Obviously, the discrepancy at phylum level suggested the difference in gut microbiota composition among six groups (Fig. 5b).

At genus level, the entire sequences of the six groups could be assigned to fourteen major genera. *Lactobacillus* was the most predominant genus in six groups, accounting for 13.00% of the total bacterial sequences. The subdominant genus *Bacteroides* and the following twelve genera were shown in Fig. 5c. DDC inducement decreased the proportion of *Lactobacillus*, *Bacteroides*, *unclassified S24*-*7*, *unclassified Lachnospiraceae*, *unclassified Prevotellaceae*, *Roseburia* and *Prevotella*, but increased the proportion of *Alistipes*, *Incertae_Sedis*, *Helicobacter*, *Parabacteroides* and *Rikenella* in MC group compared with that in NC group (Fig. 5d). The changes of unclassified *Ruminococcaceae* and *RC9_gut_group* were not obvious. While the results showed that both IOP and Qingyilidan granule had remarkable effects on gut microbiota composition. The proportions of *Lactobacillus*, *Bacteroides*, *unclassified S24*-*7*, *unclassified Lachnospiraceae*, *unclassified Prevotellaceae*, *Roseburia* and *Prevotella* were greatly increased in IOP or PC groups compared with those in MC group, but those of *Alistipes*, *Incertae_Sedis*, *Helicobacter*, *Parabacteroides* and *Rikenella* were decreased.

## Discussion

A disturbed microbiota rather than a single organism is supposed to be the pathologic agent for some chronic diseases, represented by an increase in bacterial diversity and/or an overgrowth in aggressive bacteria (Daniels et al. 2014; Walker et al. 2011). The variability of gut microbiota is possibly related to systemic inflammation. Daniels et al. found a higher diversity in diverticulitis for *Proteobacteria*, which also led to an alteration in diversity of other phyla together (Daniels et al. 2014). Administration of DDC stimulates the production of reactive oxygen species (ROS) which triggers the mitogen-activated protein kinase (MAPK)/Nuclear factor-kappa B (NF-kB) pathway and results in the production of proinflammatory cytokines, subsequently creates a hostile environment adjacent to the mucosal surface and cause a variation in gut microbiota (Silva et al. 2010; Siriwardena 2014). Although endogenous antioxidant system can prevent the toxic effect of ROS, however, excessive ROS generation caused by DDC may overwhelm the natural antioxidant defense system. Studies in mice with colitis showed that quantitative growth of *Verrucomicrobia*, *Proteobacteria* and specifically *Bacteroidetes* were almost linearly decreased as weight loss progressed (Vanhooren et al. 2013). While research also revealed that epithelium devoted to clear microbes away from mucosal surface in mounting responses and improve the proliferation of intestinal probiotics. But DDC inducement destroyed mucosal surface, which was detrimental to maintaining the stability of gut microbiota, and down-regulating the proliferation of intestinal probiotics.

In our study, IOP greatly decreased the Seq_num and OTU due to the loss of gut microbiota, which presumably provided space for normal bacteria to colonize in the gut, and a scenario also has been observed in gut microbiota variation of malnourished children in Bangladesh (Monira et al. 2011). Shannon diversity index and Chao 1 estimator showed that CP would increase the diversity and richness of mice, but IOP would decrease it. These were consistent with the study of Xu et al. that *Lentinula edodes*-derived polysaccharide can reduce the richness, diversity and evenness of microbial communities in cecum and colon (Xu et al. 2015).

In correlation analysis of the variation of gut microbiota and chemical parameters, significant correlations were found in an increase of *Bacteroidetes* and a decrease of *Firmicutes*, *Protecbacteria*, *Actinobacteria* and *Cyanobacteria*. The relative abundance of two dominant phyla, the *Bacteroidetes* and the *Firmicutes*, appears to play a role in the ability of the microbiome to harvest energy from the diet (Turnbaugh et al. 2006; Ley et al. 2005). Polsaccharides are hard to digest by human being, but recent researches have demonstrated that some microbes in the human gut can produce enzymes to hydrolyze complex polysaccharides to easily adsorbed monosaccharides (Bolam and Sonnenburg 2011). Bacteria in *Bacteroidetes* phylum may produce different kinds of polysaccharide-degrading enzymes, while *Firmicutes* phylum contains fewer microbes with the capacity of polysaccharides degradation (Bolam and Sonnenburg 2011; Ravcheev et al. 2013). Similar to other studies that *Bacteroidetes* and *Firmicutes* are predominant phyla in the gut flora (Ley et al. 2008a, b). As a dietary fungal polysaccharide, IOP has many beneficial effects on the host’s health. Oral administration of IOP may drive qualitative and selective changes in the composition of the gut microbiota (Wang et al. 2014; Wu et al. 2011). When CP mice were administrated with IOP, the proportion of *Bacteroidetes* was increased with the increasing dose of IOP, but *Firrmicutes* was decreased.

Some genera such as *Bacteroides*, *Prevotella* and *Lactobacillus* in gut microbiota play an important part in the hydrolyzation of polysaccharides into short chain fatty acids (SCFA) (Bach-Knudsen et al. 2012). Li et al. found that *Bacteroides* could produce high levels of SCFA by fermentation of indigestible plant-derived substrates in fish intestine (Li et al. 2015). Li et al. also found that *Prevotella* contained highly active hemicellulolytic and proteolytic enzymes, which could degrade polysaccharide, xylan and starch (Li et al. 2014). In the study of Reilly, oat polysaccharide increased the amount of *Lactobacilli* in intestinal microbiota and SCFA concentrations in pigs (Reilly 2010). The production of SCFA can provide energy for microbes, modulating immune responses and maintaining the epithelial barrier function (Brown et al. 2011). In our study, *Bacteroides*, *Prevotella* and *Lactobacillus* were richest in NC group but rarest in MC group, among all treatment groups, these three genera were more abundant in IOP-H group. This was consistent with the findings of Maslowski et al. that low concentrations of SCFA in germ-free mice would exacerbate inflammatory responses (Maslowski et al. 2009). An early study suggests that dietary fiber fed to conventional mice is capable to stimulate intestinal epithelial cell proliferation (Goodlad et al. 1989). In our study, IOP may also keep the integrity of epithelium, inhibit the proliferation of *Alistipes*, *Incertae_Sedis*, *Helicobacter*, *Parabacteroides* and *Rikenella* and alter the diversity of the intestinal probiotics in a dose-dependent way. However, a large number of unclassified and uncultured genera were also shown by sequence databases, the relations between CP and gut microbiota composition and how gut microbiota utilize IOP under oxidant injury need further research.

In summary, our results showed that IOP exerted pharmacological influence on CP related parameters in terms of increasing GSH-P_X_ and TAOC level and decreasing TNF-α, TGF-β, lipase and trypsin levels. Meanwhile, IOP reduced the gut microbiota diversity and richness, decreased the relative abundance of *Firmicutes*, increased the *Bacteroidetes* at phylum level, and regulated gut microbiota toward a healthy profile at genus level. Moreover, the variations in gut microbiota were correlated with biochemistry parameters. The favorable biochemical characteristics and gut microbiota suggest that IOP has strong activity for ameliorating CP and a beneficial effect on gut microbiota.

## Abbreviations

CP: chronic pancreatits
DDC: 3,5-diethoxycarbonyl-1,4-dihydrocollidine
IOP: *Inonotus obliquus* polysaccharide
GSH-P_X_: glutathione peroxidase
TAOC: total antioxidant capacity
TNF-α: tumor necrosis factor alpha
TGF-β: transforming growth factor beta
IOP-L: IOP treated group at the dosage of 0.1 g/kg/day
IOP-M: IOP treated group at the dosage of 0.2 g/kg/day
IOP-H: IOP treated group at the dosage of 0.4 g/kg/day
PC: qingyilidan granule treated group
MC: model control group
NC: normal control group
OTUs: operational taxonomic units
PLS-DA: partial least square discriminate analysis
VIP: variable importance in projection
CCA: canonical correspondence analysis
UPGMA: unweighted pair group method with arithmetic mean
PCoA: principal coordinates analysis
ROS: reactive oxygen species
MAPK: mitogen-activated protein kinase
NF-kB: nuclear factor-kappa B
SCFA: short chain fatty acids
